# Optical and morphological properties of thermochromic V_2_O_5_ coatings

**DOI:** 10.1016/j.dib.2017.07.028

**Published:** 2017-07-14

**Authors:** Sunil Kumar, Francis Maury, Naoufal Bahlawane

**Affiliations:** a Luxembourg Institute of Science and Technology (LIST), 5 avenue des Hauts-Fourneaux, L-4362 Esch-sur-Alzette, Luxembourg; b CIRIMAT, ENSIACET-4 allée E. Monso, 31030 Toulouse, France

## Abstract

We present optical and morphological characterizations performed on thermochromic V_2_O_5_ coatings. V_2_O_5_ coatings were obtained by oxidation of as-deposited VOx films. Comparisons were made among coatings oxidized at various temperatures. Photographic evidence is also shown to provide the reader a clear visual description of the color change that occurs during thermochromic process. Detailed study and analysis regarding this data can be found in Kumar et al. (2017, in press) [1,2].

## Specifications Table

**Table:** 

Subject area	*Physics, Material science*
More specific subject area	*Thermochromic oxides, chemical vapor deposition*
Type of data	*Graph, figure*
How data was acquired	1) *Total hemispherical reflection (THR) measurements were carried out on LAMBDA 1050 UV/Vis/NIR spectrophotometer from Perkin Elmer with a 150 mm integration sphere in the reflection configuration.* 2) *The film thickness and roughness were measured using an Alpha step d-500 Profilometer from KLA-Tencor* 3) *Temperature was controlled by placing the sample on a custom made heating stage with a K type thermocouple for temperature measurement and regulation.*
Data format	*Treated and analyzed*
Experimental factors	*Silicon substrates have been cleaned in Ethanol and later cut into smaller pieces before annealing at different temperatures.*
Experimental features	*Very brief experimental description*
Data source location	*Belvaux, Luxembourg*
Data accessibility	*The data are available with this article*

## **Value of the data**

• The data on visible thermochromic behavior of V_2_O_5_ coatings provides other researchers an exhaustive view of various methods used to show the thermochromic behavior.
• The data can be used by other researchers to compare and verify and improve further on the tunability of V_2_O_5_ thermochromism.
• This data will be helpful to the scientific community who wishes to use oxygen vacancy generation in metal oxides as a technique to change the optical properties

## 1 Data

**Fig. 1:**
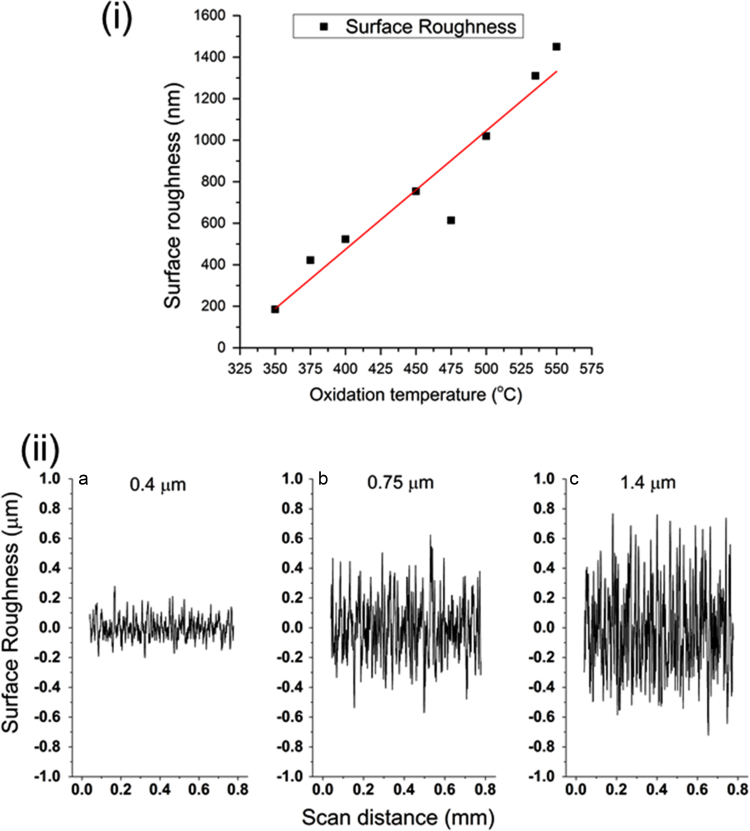
(i) Surface roughness increases linearly with oxidation temperature. (ii) Roughness profile of coatings oxidised at (a) 350 °C, (b) 450 °C and (c) 550 °C measured using a profilometer tip dragging across the surface with a scan distance of 0.8 mm.

V_2_O_5_ coatings show a linear increase in the surface roughness with temperature. Fig. 1(i) shows the plot of surface roughness versus oxidation temperature and Fig. 1 (ii) shows the roughness profile of the coatings obtained by oxidation at (a) 350 °C, (b) 450 °C and (c) 550 °C over a scanning distance of 0.8 mm.

**Fig. 2:**
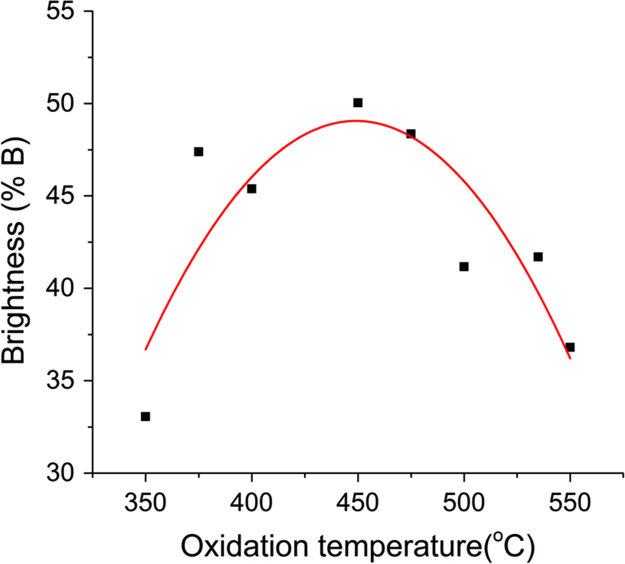
Brightness versus oxidation temperature curve indicates a maximum brightness at 450 °C. It is noteworthy that sample colour is bright yellow at this oxidation temperature.

The brightness of the coatings is defined as the total area under the curve over the full range of visible spectrum (400–800 nm). The curve plotted with brightness versus oxidation temperature shown in Fig. 2 has a bell curve profile with maximum at 450 °C and a brightness of 50%.

**Fig. 3:**
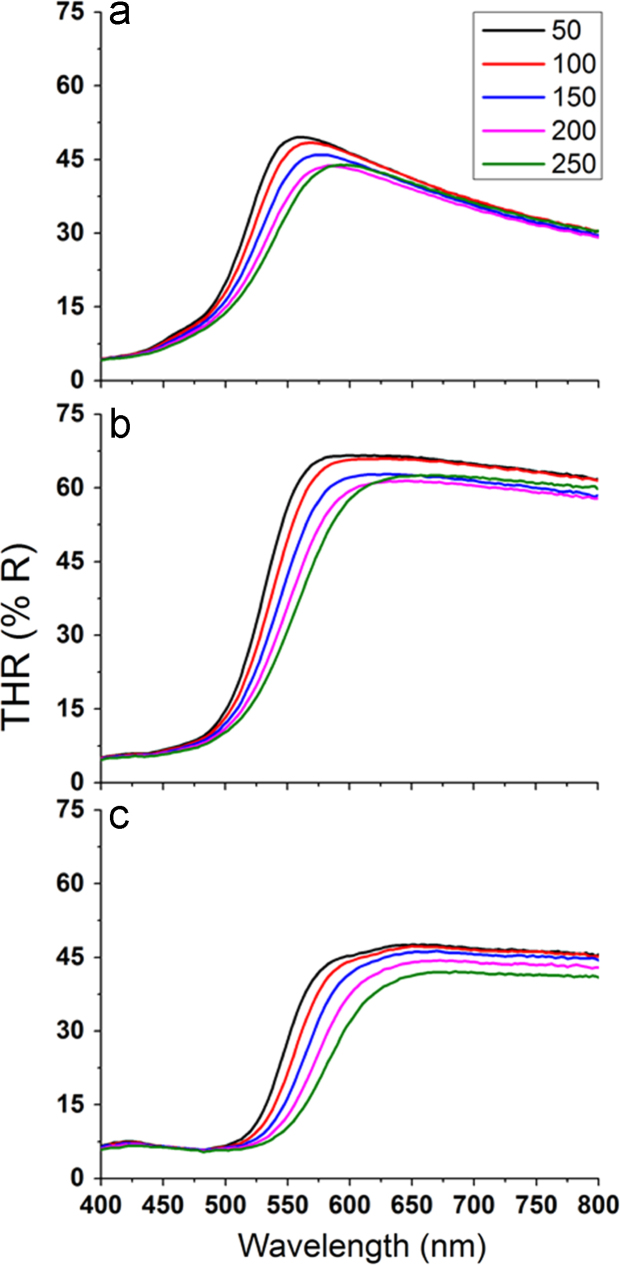
Temperature dependent optical spectra of coatings obtained by oxidation at (a) 350 °C, (b) 450 °C and (c) 550 °C respectively.

Temperature-dependent optical spectra in the visible region are shown in Fig. 3 for coatings obtained by oxidation at (a) 350 °C, and (b) 450 °C and (c) 550 °C respectively.

**Fig. 4:**
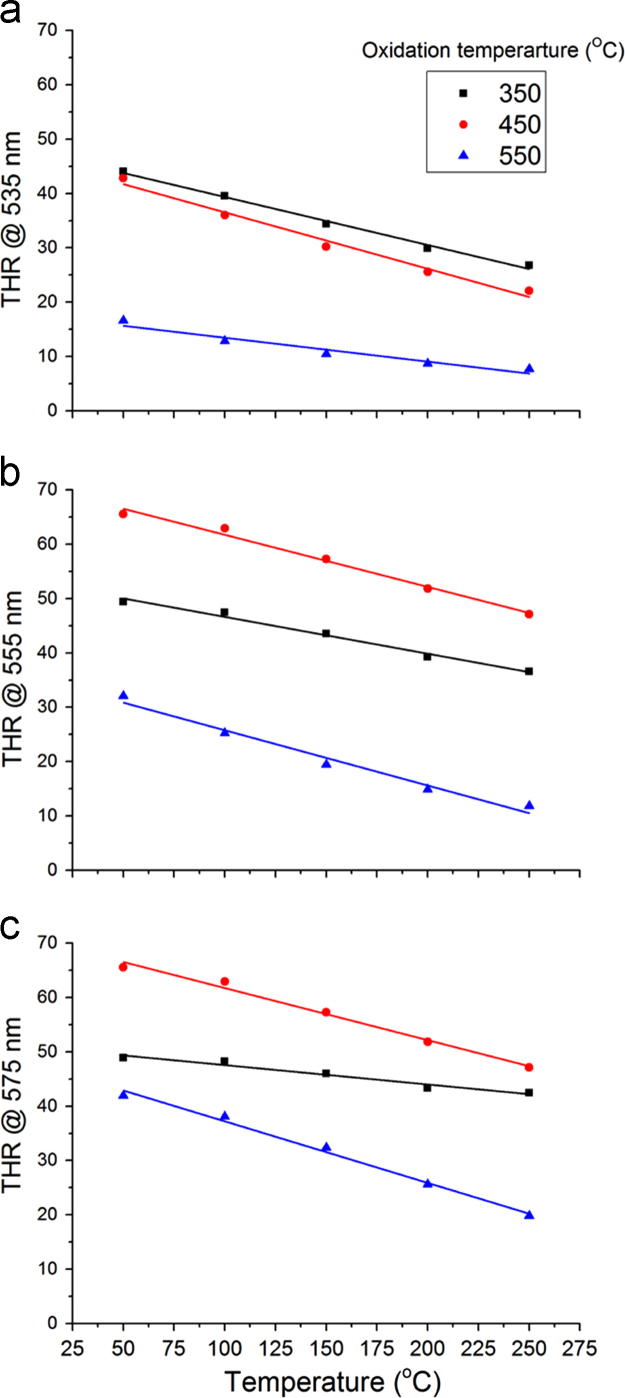
Plot of THR at (a) 535 nm, (b) 555 nm and (c) 575 nm versus temperature for V_2_O_5_ coatings annealed at 350 °C, 450 °C and 550 °C respectively.

**Fig. 5:**
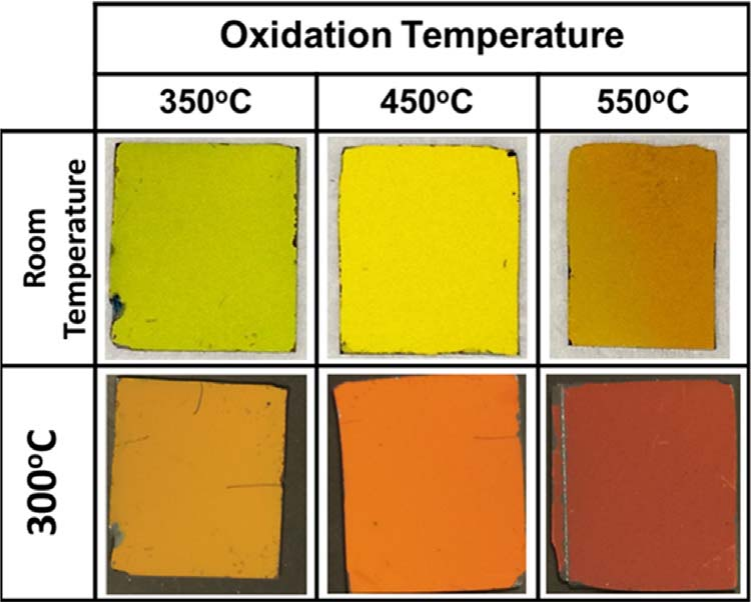
Photographs of V_2_O_5_ coatings on silicon wafer, obtained by oxidation at different temperatures. Thermochromic colour change for each film is shown upon heating the films from room temperature (1st row) till 300 °C (2nd row).

THR at specific wavelengths like 535 nm, 555 nm and 575 nm was compared among the films obtained by oxidation at 350 °C, 450 °C and 550 °C in Fig. 4. Lastly Fig. 5 shows the photographic images of thermochromic V_2_O_5_ coatings at both room temp and 300 °C

## 2 Experimental design, materials and methods

### 2.1 Preparation of V_2_O_5_ coatings

Thin films of vanadium oxide were deposited on silicon substrates by Direct Liquid Injection (DLI) Metal Organic Chemical Vapor Deposition (MOCVD), the details of which are reported elsewhere [1], [2]. Argon was used as the carrier gas at a flow rate of 50 sccm while the chamber pressure was adjusted to 10 mbar. Substrates were maintained at a constant temperature of 500 °C during the four hours of deposition.

After deposition, samples were allowed to cool till room temperature in argon atmosphere at low pressure before withdrawing from the chamber. Further handling of the samples was carried out under ambient atmosphere. Post deposition annealing was performed under ambient air at 300–580 °C. The annealing time was adjusted to allow a complete oxidation from VOx to V_2_O_5_. While 10 min were sufficient for oxidation at 550 °C, significantly longer times were required at lower temperatures; this can be explained by simple temperature dependent oxidation kinetics.

To isolate V_2_O_5_ coatings form atmospheric gas phase interactions, Atomic layer deposition (ALD) of Al_2_O_3_ was performed using the sequential introduction of Trimethylaluminium (TMA) and water. The pulse times for each reactant were adjusted to 40 ms with a 15 s purge in between each pulse. The rather large pulse and purge times were chosen to achieve complete conformal coverage over the film.

### 2.2 Film characterization

Total hemispherical reflection (THR) measurements were carried out on LAMBDA 1050 UV/Vis/NIR spectrophotometer from Perkin Elmer with a 150 mm integration sphere in the reflection configuration. Measurements, which correspond to the sum of specular and diffuse reflections, were performed in the visible spectral range (400–800 nm). Temperature-dependent measurements were carried out with the help of a custom made sample holder with an integrated heating element. Temperature control was achieved by a Horst HT 60 temperature controller coupled to a K-type thermocouple. The film thickness and roughness were measured using an Alpha step d-500 Profilometer from KLA-Tencor.

## Transparency document Supplementary material

Supplementary material.
