# Supplementary figures and images for: Optical and morphological properties of thermochromic V2O5 coatings
Source: Data Brief. 2017 Jul 14;14:348–53. doi: 10.1016/j.dib.2017.07.028 (PMC5545823; doi:10.1016/j.dib.2017.07.028)

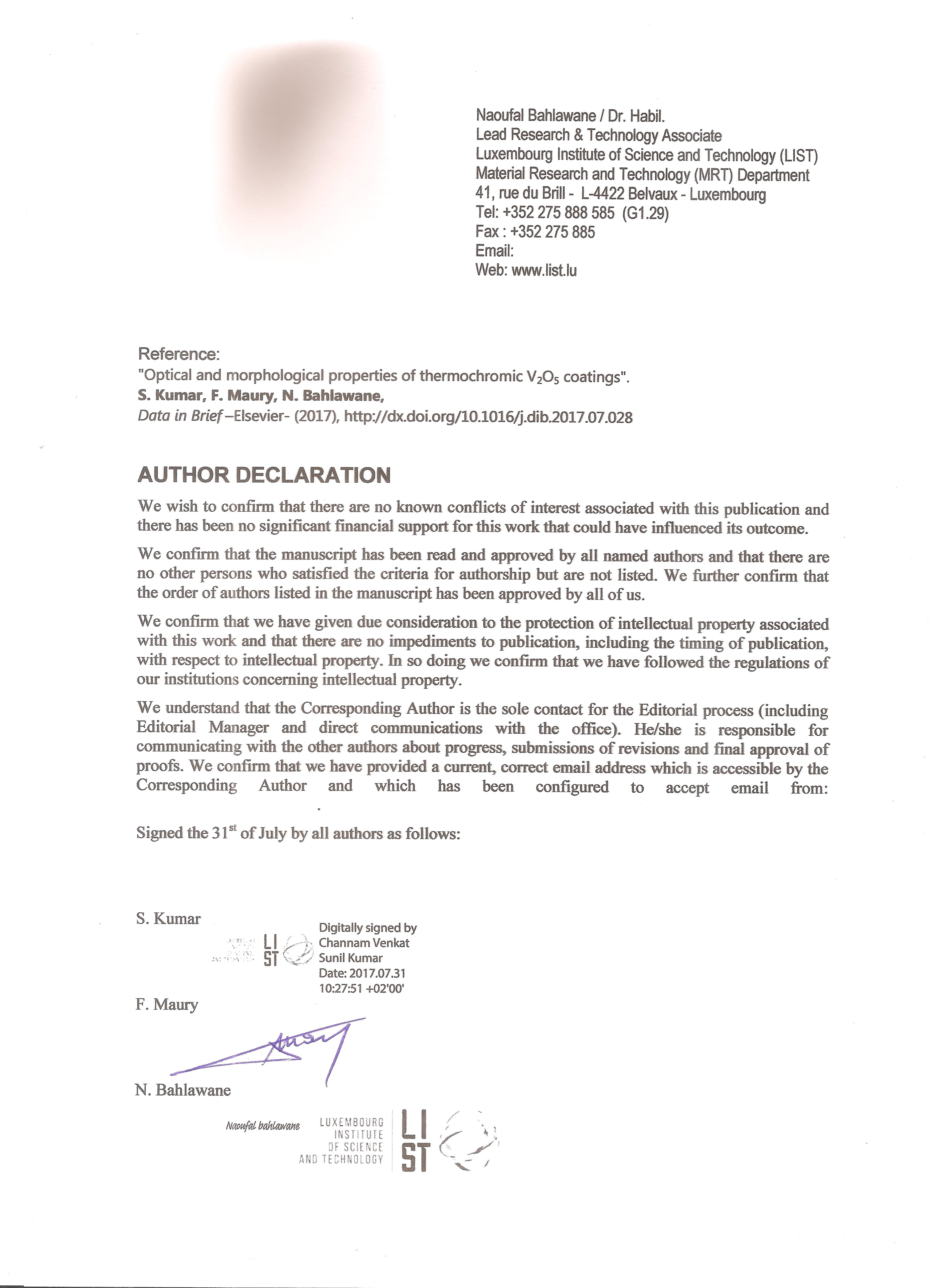

Supplement: Supplementary file 1 — Supplementary material [file mmc1.jpg]
